# Household income unequally affects genetic susceptibility to pulmonary diseases: evidence from bidirectional Mendelian randomization study

**DOI:** 10.3389/fmed.2024.1279697

**Published:** 2024-07-04

**Authors:** Hongfa Xu, Hailian Deng, Yueying Wu, Yu Yang, Xifeng Zheng

**Affiliations:** ^1^Department of Ontology, Affiliated Hospital of Guangdong Medical University, Zhanjiang, China; ^2^Department of Geriatrics, Affiliated Hospital of Guangdong Medical University, Zhanjiang, China; ^3^Department of Cardiology, Affiliated Hospital of Guangdong Medical University, Zhanjiang, China

**Keywords:** household income status, pulmonary diseases, causal relationship, instrumental variable, Mendelian randomization

## Abstract

**Objectives:**

Previous observational studies have reported a close association between socioeconomic status and pulmonary disease-related morbidity. However, the inherent causal effects remain unclear. Therefore, this bidirectional Mendelian randomization (MR) study aimed to identify the causal relationship between household income and genetic susceptibility to pulmonary diseases.

**Methods:**

An MR study was conducted on a large cohort of European individuals, using publicly available genome-wide association study datasets using a random-effects inverse-variance weighting model as the main standard. Simultaneously, MR-Egger regression, weighted median, and maximum likelihood estimation were applied as supplements. Sensitivity analysis, comprising a heterogeneity test and horizontal pleiotropy test, was performed using the Cochran’s Q, MR-Egger intercept, and MR-PRESSO tests to ensure the reliability of the conclusion.

**Results:**

A higher household income tended to lower the risk of genetic susceptibility to chronic obstructive pulmonary disease (COPD, OR: 0.497, 95% CI = 0.337–0.733, *p* < 0.001), asthma (OR: 0.687, 95% CI = 0.540–0.876, *p* = 0.002), and lung cancer (OR: 0.569, 95% CI = 0.433–0.748, *p* < 0.001), and further indicated potential causality with pneumonia (OR: 0.817; 95% CI = 0.686–0.972, *p* = 0.022). No association was evident with COVID-19 (OR: 0.934, 95% CI = 0.764–1.142, *p* = 0.507), tuberculosis (OR: 0.597, 95% CI = 0.512–1.189, *p* = 0.120), or bronchiectasis (OR: 0.680, 95% CI = 0.311–1.489, *p* = 0.400). Reverse MR analysis suggested no reverse causal relationship between pulmonary disease and household income status, while sensitivity analysis verified the reliability of the results.

**Conclusion:**

The results revealed that the population with a higher household income tended to have a lower risk of genetic susceptibility to COPD, asthma, and lung cancer.

## 1 Introduction

The 2017 Global Burden of Disease Study reported that chronic pulmonary diseases are among the primary causes of mortality, impacting approximately 600 million individuals globally, with four million dying prematurely from these diseases. Compared to the results obtained in 1990, the morbidity and mortality rates have increased by 39.8 and 18.0%, respectively (1). Chronic obstructive pulmonary disorder (COPD), asthma, acute respiratory infections (especially the coronavirus disease 2019 [COVID-19] pandemic), and lung cancer are the most common pulmonary diseases which together place a significant financial burden on global public health. Numerous observational studies and meta-analyses have shown that socioeconomic status is closely linked to pulmonary disease-related morbidity (2). For example, one repeated cross-sectional analysis of 160,495 participants spanning half a century in America reported that socioeconomic disparities in pulmonary health have persisted, and potentially worsened over the past six decades. Socioeconomic status can serve as an independent determinant of pulmonary health (3), a perspective which was also supported by independent studies conducted in China and Europe (4, 5). Nevertheless, observational studies have limitations, such as the absence of randomization, potential confounding factors, challenges in controlling variables, and an inability to establish causality due to unaccounted factors that may bias the outcomes. Thus, additional research is necessary to gain a deeper understanding of this relationship.

Mendelian randomization (MR) utilizes valuable genetic variants, such as single nucleotide polymorphisms (SNPs), as instrumental variables (IVs) to evaluate the causal effects between modifiable, non-genetic exposure factors and genetic susceptibility to diseases. This type of analysis is grounded in Mendel’s law of inheritance, which explains the random allocation of genetic variants during meiosis (6). In the absence of randomized controlled trials (RCTs), MR studies offer an alternative approach for causal inference, as genetic variants are randomly assigned during meiosis, thus mimicking the randomization in the RCT process. Compared to traditional observational studies, the main advantage of MR studies is that they are less likely to be influenced by unmeasured confounding factors as genetic variants are identified at the time of conception (7, 8). MR studies have successfully elucidated causal relationships between behavioral exposure, education, socioeconomic conditions, and diseases (9–11). Therefore, the aim of this study was to identify a bidirectional causal relationship between household income and genetic susceptibility to common pulmonary diseases using MR.

## 2 Materials and methods

### 2.1 Study design and information of GWAS datasets

To achieve impartial results, an MR study depends on three fundamental assumptions: (1) the selected genetic IVs must be significantly associated with the exposure factor; (2) the IVs should be independent of potential confounders associated with exposure factors and outcomes; and (3) the IVs should affect the outcomes only through the exposure factor (7).

This study was based on a large sample cohort of European individuals extracted from publicly available genome-wide association study (GWAS) datasets. The variable genetic information used in this study was extracted from the Integrative Epidemiology Unit (IEU) GWAS database^1^ (12), a publicly available GWAS summary database. The GWAS summary dataset contains data on the “average total household income before tax,” encompassing the household income status of 397,751 samples originally from the UK biobank database. Annual household income was divided into five intervals: >100,000 (in pounds), 52,000–100,000, 31,000–51,999, 18,000–30,999, and <18,000. Pulmonary diseases encompass a range of conditions, including chronic obstructive pulmonary disease (COPD), asthma, tuberculosis, bronchiectasis, COVID-19, pneumonia, and lung cancer. Amongst which, the data of COPD, asthma, tuberculosis, bronchiectasis, and pneumonia were collected from the FinnGen consortium. The cases were identified using the International Classification of Diseases (ICD). For further information, please refer to https://r10.risteys.finngen.fi/. For COVID-19 cases, individuals must have laboratory-confirmed SARS-CoV-2 infection (RNA and/or serology-based), EHR/ICD coding indicating COVID-19, or self-reporting of a positive COVID-19 test (e.g., via questionnaire). We followed sample size and timeliness priorities to make the best possible choice. Detailed information on all the GWAS datasets is listed in Table 1. The GWAS datasets of household income status and pulmonary diseases were originated from different consortiums to decrease potential bias caused by sample overlap. In addition, to minimize racial mismatches, all GWAS datasets involved in this study majorly enrolled populations of European ancestry. This study involved 14 times MR analyses to explore the bidirectional association between annual household income status and seven different pulmonary diseases.

**Table 1:** Basic information of the GWAS datasets utilized in this study.

Traits	GWAS ID	Year	Population	Sample size
Exposure factor				Total sample
Household income status (13)	ukb-b-7408	2018	European	397,751
Outcomes				Case/Control
COPD (14)	finn-b-J10_COPD	2021	European	6,915/186,723
Asthma (14)	finn-b-J10_ASTHMA	2021	European	20,629/135,449
Tuberculosis (14)	finn-b-AB1_TUBERCULOSIS	2021	European	1,193/217,599
Bronchiectasis (14)	finn-b-J10_BRONCHIECTASIS	2021	European	1,107/186,723
COVID-19 (15)	ebi-a-GCST011073	2020	European	38,984/1,644,784
Pneumoniae (14)	finn-b-J10_PNEUMONIA	2021	European	27,370/191,422
Lung cancer	ieu-a-987	–	European	29,863/55,586

### 2.2 Selection criteria for IVs

SNPs filtered according to the three pivotal MR assumptions were used as IVs. First, SNPs were matched using a genome-wide statistical significance threshold (*p* < 5 × 10^−8^). Second, the corresponding linkage disequilibrium state, as well as the independence of these SNPs in the linkage disequilibrium state and the independence of these SNPs within a 0–10,000 kb window at a threshold of *r*^2^ < 0.001. Third, to evaluate the assumption that the IVs affect the outcomes only through the exposure factor, potential phenotypes that may be relevant to the IVs were investigated by searching the human genotype–phenotype association database (PhenoScanner-V2, http://www.phenoscanner.medschl.cam.ac.uk/) (16). Fourth, the SNPs identified as IVs were further matched with those in the outcome GWAS dataset to establish genetic associations. The summary SNP phenotype and outcome statistics were harmonized to ensure effect size alignment, while palindromic SNPs were excluded. Finally, *F*-statistics (>10) were applied to evaluate the strength of the IVs to avoid the influence of weak instrumental bias (17).

### 2.3 Mendelian randomization study and sensitivity analysis

The MR study was performed using a random-effects inverse-variance weighting (IVW) model (18) as the primary standard and three other models (MR-Egger regression (19), weighted median (20) and maximum likelihood (21)) as supplements to evaluate the potential causal relationship between household income status and seven pulmonary diseases. A reverse MR study evaluated the potential causal relationship between seven pulmonary diseases and household income status using the same methods. In addition, a sensitivity analysis was performed to measure the reliability and stability of the conclusion. The sensitivity analysis comprised: (1) Cochran’s Q test (according to the IVW or MR-Egger regression model), (2) horizontal pleiotropy test using MR-Egger intercept (22) and MR-PRESSO test (23), and (3) “leave-one-out” test (in which each SNP was abandoned successively to repeat the IVW analysis to identify whether any specific SNP drives the causal relationship estimate). The results are reported as odds ratios (OR) with corresponding 95% confidence intervals (CI) and *p*-values and are illustrated as scatter plots. The evidential threshold in the MR analysis was defined as *p*-value <0.004 (0.05/14) according to the Bonferroni correction method. A *p*-value <0.05, but above the Bonferroni-corrected evidential threshold, was considered to indicate a potential association. For sensitivity analysis, statistical significance was set at *p*-value <0.05. The “TwoSampleMR” (24) and “MR-PRESSO” (23) packages of R 4.0.3 software were used to process and visualize the data.

## 3 Results

### 3.1 Results of the MR study

Forty-three SNPs were identified as IVs in each outcome dataset. The *F*-statistic score of all the selected SNPs was >10 (COPD: 57.64, asthma: 57.87, tuberculosis: 57.77, bronchiectasis: 57.77, COVID-19: 57.77, pneumonia: 57.49, and lung cancer: 57.76), indicating a low risk of weak instrumental bias.

According to the results of random-effects IVW model with Bonferroni correction as the primary standard, a higher household income tended to lower the risk of genetic susceptibility to COPD (OR: 0.497, 95% CI = 0.337–0.733, *p* < 0.001), asthma (OR: 0.687, 95% CI = 0.540–0.876, *p* = 0.002), and lung cancer (OR: 0.569, 95% CI = 0.433–0.748, *p* < 0.001), and indicated a potential causality with pneumonia (OR: 0.817, 95% CI = 0.686–0.972, *p* = 0.022). However, no evidence was obtained to suggest a potential causal relationship between household income status and COVID-19 (OR: 0.934, 95% CI = 0.764–1.142, *p* = 0.507), tuberculosis (OR: 0.597, 95% CI = 0.512–1.189, *p* = 0.120), or bronchiectasis (OR: 0.680, 95% CI = 0.311–1.489, *p* = 0.400). The results of the weighted median and maximum likelihood estimation models supported these conclusions. Unfortunately, the MR-Egger regression model results did not show any statistically significant differences.

**Figure 1:**
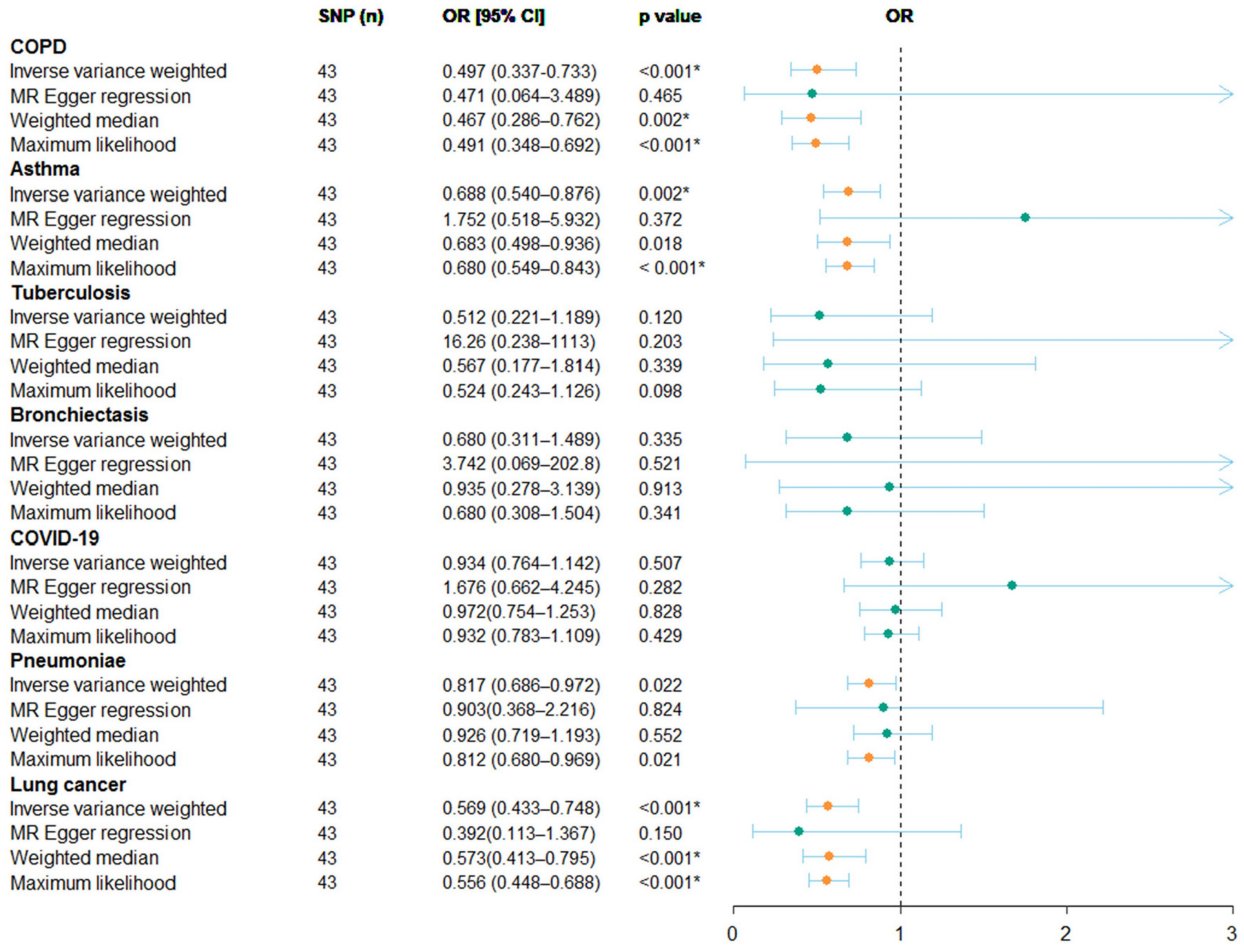
Result of the MR study illustrated as a forest plot. The causal relationship between household income status and pulmonary diseases by MR study is shown. OR, odds ratio; CI, confidence interval.

In summary, the MR study revealed that the population with superior household income tended to have a lower genetic susceptibility risk to COPD, asthma, and lung cancer. Detailed information is displayed in the forest map in Figure 1, and as a scatter plot in Supplementary Figure S1.

### 3.2 Results of sensitivity analyses in the MR study

The results of Cochran’s Q test indicated a certain degree of heterogeneity among the IVs in lung cancer (Table 2), but not in the other six types of pulmonary diseases. Thus, the random-effects IVW model was used to minimize the effect of heterogeneity in the MR study to the greatest possible extent. More importantly, no horizontal pleiotropy was detected using the MR-Egger intercept or MR-PRESSO tests (Table 2). In addition, the “leave-one-out” method indicated that no specific SNP among the IVs significantly affected the overall results (Supplementary Figure S2). In summary, the sensitivity analyses verified the robustness of the conclusions.

**Table 2:** Results of heterogeneity and horizontal pleiotropy tests.

Diseases	Heterogeneity test		Horizontal pleiotropy test	
	MR-Egger regression	IVW model	MR-Egger intercept	MR-PRESSO test
COPD	0.063	0.077	0.957	0.073
Asthma	0.110	0.081	0.133	0.068
Tuberculosis	0.177	0.130	0.110	0.195
Bronchiectasis	0.536	0.547	0.398	0.468
COVID-19	0.060	0.051	0.214	0.059
Pneumoniae	0.429	0.471	0.824	0.571
Lung cancer	0.002	0.003	0.553	0.446

IVW, inverse variance weighting. *p*-value < 0.05 was considered to indicate statistical differences in both heterogeneity and horizontal pleiotropy tests.

### 3.3 Results of reverse MR study and sensitivity analyses

The number of SNPs that were ultimately identified as IVs in different pulmonary diseases in the reverse MR study were 15 for asthma, 13 for lung cancer, 7 for COVID-19, 3 for COPD, 1 for bronchiectasis and pneumonia, and 0 for tuberculosis. Based on the results of random-effects IVW model with Bonferroni correction as the primary standard, the reverse MR study suggested no reverse causal relationship between pulmonary disease and household income. Detailed information is displayed in Table 3.

**Table 3:** Results of the reverse MR study.

Disease	SNPs (*n*)	OR (95% CI)	*p*-value
COPD
Inverse variance weighted	3	0.994 (0.946–1.046)	0.826
MR Egger regression	3	1.144 (1.058–1.238)	0.184
Weighted median	3	0.987 (0.964–1.011)	0.281
Maximum likelihood	3	0.994 (0.974–1.014)	0.552
Asthma
Inverse variance weighted	15	0.984 (0.963–1.005)	0.144
MR Egger regression	15	0.925 (0.833–1.026)	0.165
Weighted median	15	0.977 (0.956–0.999)	0.039
Maximum likelihood	15	0.984 (0.968–1.000)	0.053
Tuberculosis
Inverse variance weighted	0	NA	NA
MR Egger regression	0	NA	NA
Weighted median	0	NA	NA
Maximum likelihood	0	NA	NA
Bronchiectasis
Inverse variance weighted	1	NA	NA
MR Egger regression	1	NA	NA
Weighted median	1	NA	NA
Maximum likelihood	1	NA	NA
Wald ratio method	1	1.027 (1.007–1.047)	0.006
COVID-19
Inverse variance weighted	7	0.986 (0.960–1.013)	0.318
MR Egger regression	7	0.966 (0.884–1.056)	0.479
Weighted median	7	0.984 (0.950–1.019)	0.357
Maximum likelihood	7	0.986 (0.960–1.013)	0.312
Pneumoniae
Inverse variance weighted	1	NA	NA
MR Egger regression	1	NA	NA
Weighted median	1	NA	NA
Maximum likelihood	1	NA	NA
Wald ratio method	1	1.099 (1.006–1.200)	0.035
Lung cancer
Inverse variance weighted	13	0.985 (0.962–1.008)	0.193
MR Egger regression	13	0.986 (0.935–1.039)	0.610
Weighted median	13	0.983 (0.964–1.002)	0.079
Maximum likelihood	13	0.984 (0.971–0.998)	0.022

The causal relationship between pulmonary diseases and household income status by reverse MR study. OR, odds ratio; CI, confidence interval; NA, none available.

## 4 Discussion

Previous epidemiological studies have demonstrated that socioeconomic status has a significant impact on pulmonary diseases (25). Given its integral nature as an aspect of socioeconomic status, household income status has also consistently been linked to pulmonary diseases. Nevertheless, the underlying causal relationship remains unclear. To the best of our knowledge, this study is the first to investigate the causal relationship between household income status and pulmonary diseases using a bidirectional two-sample MR approach. Our findings suggest that individuals with a higher household income tend to have a lower risk of developing COPD, asthma, and lung cancer. Additionally, a reverse MR analysis did not indicate any reverse causal relationship between pulmonary disease and household income status.

Our findings are supported by accumulating evidence from both cohort studies and meta-analyses. Adeloye et al. reported that more than three-quarters of global COPD cases occur in low- and middle-income countries (26). Meanwhile, a multicenter study with 11,042 participants directed by Matthew Grigsby revealed that the prevalence of COPD was negatively associated with monthly household income status (OR = 0.96 per category, 95% CI = 0.93–0.99) (27). Moreover, Pakhale et al. reviewed the association between socioeconomic status and COPD morbidity in Ottawa. They showed that the population with a lower household income had a significantly higher COPD prevalence than the average citizen (28). Similarly, Yan Borné et al. suggested that, in Sweden, low annual income is a risk factor for COPD (hazard ratio [HR] = 2.23; 95% CI = 1.97–2.53, *p* < 0.001) (29). Asthma is another threatening respiratory disease affecting all age groups, particularly children (30). Multiple observational studies have concluded that populations with inferior family income status are at an increased risk of suffering from asthma (31–33). In addition, lung cancer is an increasing problem in developing countries because of increasing smoking trends, high incidence of air pollution, lack of awareness and screening, delayed presentation, and late diagnosis (34). In this study, we identified low-income status as a risk factor for lung cancer. This viewpoint concurs with the conclusions drawn by Orlewska et al. in Poland (35) and Fabian Tetzlaff et al. in Germany (36). Additionally, a prior study on the geographical distribution of mortality in urban areas in Spain revealed that areas with high mortality from COPD and lung cancer often coincided with low-income neighborhoods (37). Together, the results of these studies show that household income status and the poor living environment which occurs as a consequence are risk factors for respiratory diseases, such as COPD, asthma, and lung cancer.

Several pathological mechanisms may explain the association between household income and genetic susceptibility to pulmonary diseases. First, it is well known that air pollution and smoking are essential risk factors for pulmonary diseases. Populations with inferior household income status and living standards in low-income countries may not only be susceptible to poor living conditions, but may also be more likely engage in unhealthy lifestyle behaviors such as smoking. Gaffney et al. reported that, in the United States, current/former smoking rates in the highest income quintile decreased by ~50% from 1971 to 2018, while the rates in the lowest income quintile remained largely unchanged at an alarming level of ~60% (3). Secondly, populations with lower household incomes are more likely to face deficiencies in nutrients, such as high-quality protein, fresh fruits and vegetables, dietary fiber, vitamins, and micronutrients. These nutrients are closely linked to immune system function *in vivo*, particularly during pregnancy and childhood (38–41). Finally, low-income populations are less likely to have access to healthcare services and more likely to lack health consciousness, which may result in respiratory diseases that persist for a long time. Gaffney et al. concluded that, even though the uninsured rate among adults with asthma in the US decreased from 19.4 to 9.6% (adjusted 9.27 percentage points; 95% CI = 7.1–11.5%) from 1997 to 2018, the proportions delaying or foregoing medical care because of cost or going without medications did not improve. Meanwhile, in the same study, a similar trend was also reported in patients with COPD (42).

Although this study has provided valuable scientific insights, however, there are several limitations that need to be addressed. Firstly, the GWAS datasets utilized in the MR analysis primarily including individuals of European descent, suggesting that the findings may not be fully applicable to other ethnic groups. Secondly, the biological mechanisms of SNPs and their impact on human susceptibility to respiratory diseases require further exploration. Finally, the current MR study did not include a sub-group analysis for populations known to be at increased risk of COPD, asthma, and lung cancer. By incorporating such an analysis, we could enhance the practical utility of the study by obtaining a more comprehensive understanding of how household income affects diverse population subgroups. This, in turn, could facilitate the development of more targeted policies and support measures.

## 5 Conclusion

Overall, the results of this bidirectional MR study demonstrated that individuals with higher household income exhibit a reduced risk of genetic predisposition to COPD, asthma, and lung cancer. These findings highlight the need to include household income disparities in medical reimbursement policies, and to prioritize efforts to enhance equal access to and availability of medical services for individuals in low-income households.

## Data availability statement

The original contributions presented in the study are included in the article/Supplementary material, further inquiries can be directed to the corresponding author.

## Ethics statement

The requirement of ethical approval was waived for the studies involving humans because the variable genetic information involved in this study was extracted from the Integrative Epidemiology Unit (IEU) GWAS database (12) (https://gwas.mrcieu.ac.uk/), which is a publicly available GWAS summary database. The studies were conducted in accordance with the local legislation and institutional requirements. The participants provided their written informed consent to participate in these studies.

## Author contributions

HX: Conceptualization, Writing – original draft. HD: Writing – original draft, Data curation. YW: Data curation, Writing – original draft. YY: Conceptualization, Supervision, Writing – review & editing. XZ: Methodology, Project administration, Writing – review & editing.

## Footnotes

^1^https://gwas.mrcieu.ac.uk/
