# Supplementary figures and images for: Household income unequally affects genetic susceptibility to pulmonary diseases: evidence from bidirectional Mendelian randomization study
Source: Front Med (Lausanne). 2024 Jul 4;11:1279697. doi: 10.3389/fmed.2024.1279697 (PMC11254668; doi:10.3389/fmed.2024.1279697)

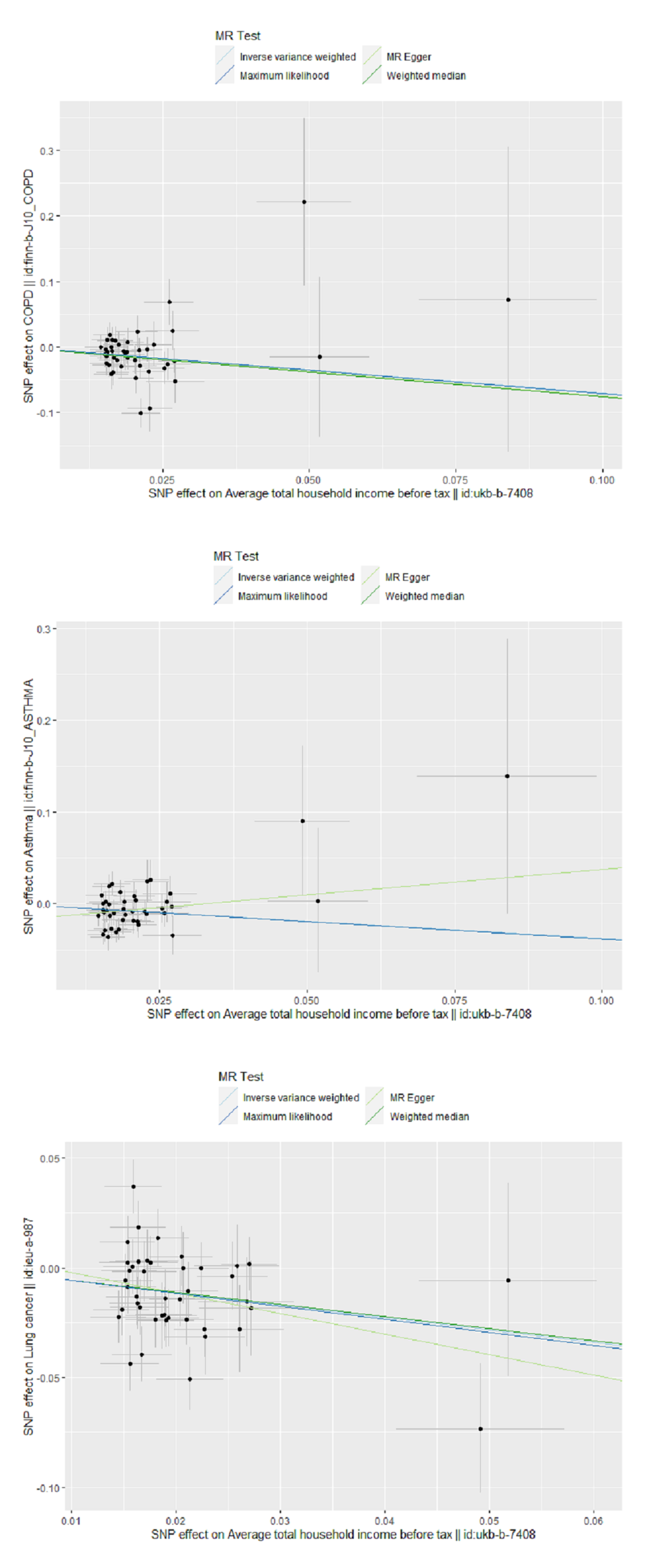

Supplement: Supplementary file 1 [file Image_1.PNG]

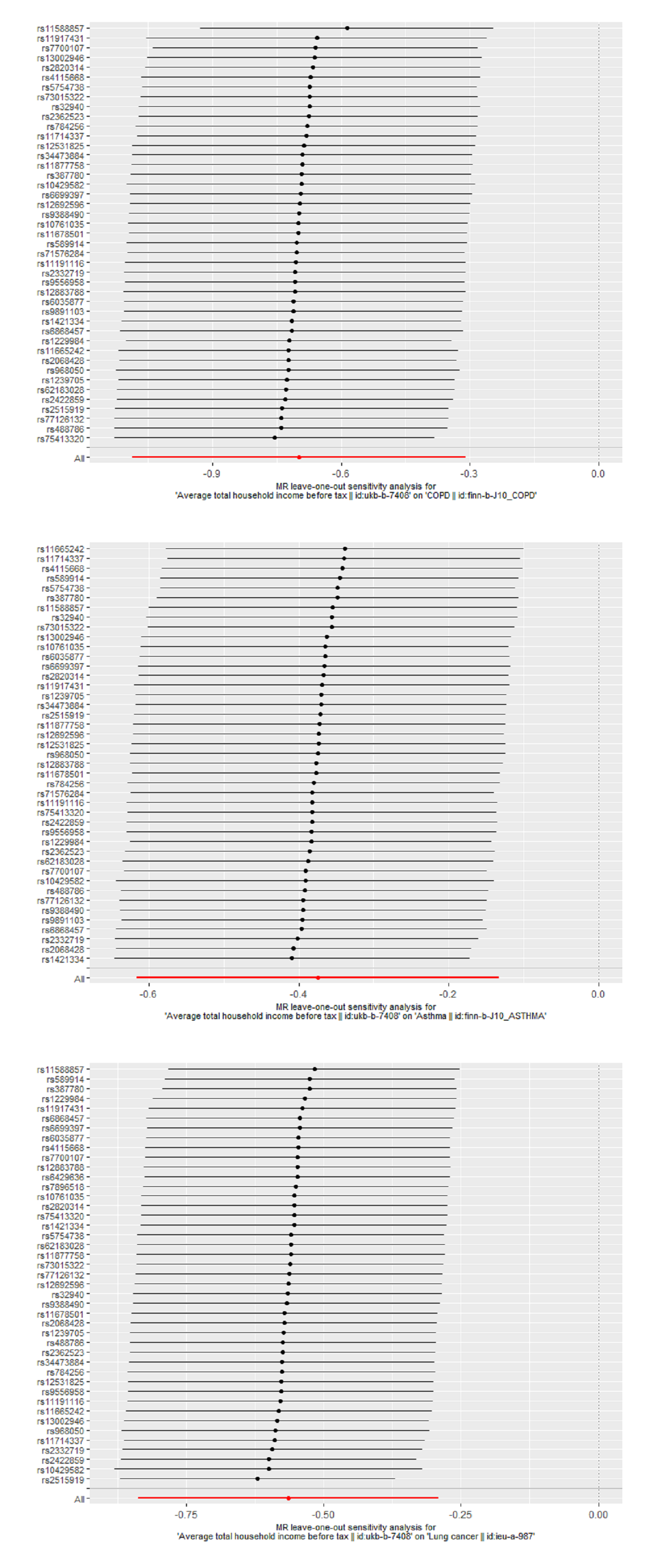

Supplement: Supplementary file 2 [file Image_2.PNG]
